# The SPB-Box Transcription Factor *AaSPL2* Positively Regulates Artemisinin Biosynthesis in *Artemisia annua* L.

**DOI:** 10.3389/fpls.2019.00409

**Published:** 2019-04-09

**Authors:** Zongyou Lv, Yun Wang, Yan Liu, Bowen Peng, Lei Zhang, Kexuan Tang, Wansheng Chen

**Affiliations:** ^1^Department of Pharmacy, Changzheng Hospital, Second Military Medical University, Shanghai, China; ^2^Research and Development Center of Chinese Medicine Resources and Biotechnology, Shanghai University of Traditional Chinese Medicine, Shanghai, China; ^3^Department of Pharmacy, Xinhua Hospital, Shanghai Jiao Tong University School of Medicine, Shanghai, China; ^4^Joint International Research Laboratory of Metabolic and Developmental Sciences, Key Laboratory of Urban Agriculture (South) Ministry of Agriculture, Plant Biotechnology Research Center, Fudan-SJTU-Nottingham Plant Biotechnology R&D Center, Shanghai Jiao Tong University, Shanghai, China; ^5^Department of Pharmaceutical Botany, School of Pharmacy, Second Military Medical University, Shanghai, China

**Keywords:** SPL transcription factor, jasmonic acid, transcript regulation, artemisinin, *Artemisia annua*

## Abstract

Artemisinin, an important compound produced by *Artemisia annua*, is the active ingredient in the treatment of malaria. Jasmonic acid, one of the phytohormones, is an important elicitor of artemisinin biosynthesis by enhancing transcription levels of transcription factors. SPL transcription factors are plant-specific transcription factors of plant growth, development, and secondary metabolism regulation. However, to date, the SPL transcription factors that regulate artemisinin biosynthesis is currently unclear. Here, we show that an SPL transcription factor can positively regulate artemisinin biosynthesis by binding to the promoter of artemisinin biosynthetic pathway genes. We screened *AaSPL2* by gene expression profiles analysis in 14 SPL transcription factors. We demonstrated that AaSPL2 can activate the promoter of *DBR2* by dual-LUC assy. Moreover, in the *AaSPL2* overexpression plants, the artemisinin content was increased by 33–86%, and in the *AaSPL2* -RNAi transgenic plants, artemisinin content was decreased by 33–65%. These data suggest that *AaSPL2* and *DBR2* interact with a “GTAC” *cis*-element in the *DBR2* promoter, mediating the transcriptional activation of *DBR2* in response to JA and resulting in the improvement on artemisinin content.

## Introduction

**FIGURE 1:**
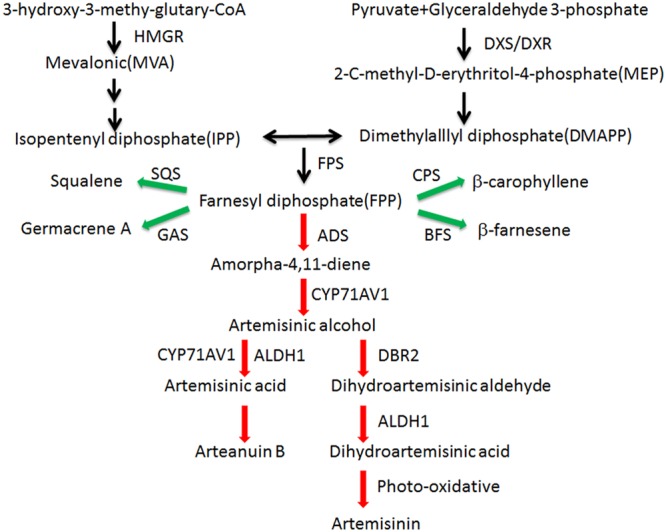
Artemisinin biosynthetic pathways in *A. annua*. Artemisinin derived from MVA and MEP pathways. Enzymes marking in red are the artemisinin biosynthetic pathway genes and enzymes marking in green are the artemisinin biosynthetic branch-pathway genes. ADS: amorpha-4,11-diene synthase (AF138959); ALDH1: aldehyde dehydrogenase 1 (FJ809784); BFS: β-farnesene synthase (AY835398); CPS:β-caryophyllene synthase (AF472361); CYP71AV1: amorphadiene-12-hydroxylase (DQ453967); DBR2: artemisinic aldehyde Δ11(13) reductase (EU704257); DXR: 1-deoxy-Dxylulose-5-phosphate reductoisomerase (AF182287); DXS: 1-deoxy-D-xylulose-5-phosphate synthase (AF182286); FPS: farnesyl diphosphate synthase (U36376); GAS: germacrene A synthase (DQ447636); HMGR:3-hydroxy-3-methyl-glutaryl coenzyme A reductase (AF142473); SQS: squalene synthase (AY445505).

Malaria is a global infectious disease that 3.3 billion people are easy to infect it. Artemisinin combination therapy (ACT) has been the frontline treatment for malaria in recent years (World Health Organization, 2014). Artemisinin is produced in the glandular trichomes of *Artemisia annua* L (Duke and Paul, 1993). The artemisinin biosynthetic pathway is well studied (Figure 1). In recent years, the semisynthesis of artemisinin following microbial production of artemisinic acid has already succeeded (Paddon et al., 2013). Because the price of artemisinin is fluctuating, the microbial deriving artemisinin may supplement the market but not replace agricultural production (Peplow, 2016). Our aim is to improve the content of artemisinin and reduce production costs.

In general, the average content of *A. annua* lies between 0.5–1.2%, while the purification yields are typically in the range of 50–80% of the crude extract (Kindermans et al., 2007). Because of the short supply and high costs of artemisinin, some strategies are employed to increase artemisinin production, such as blocking branch pathway genes (Zhang et al., 2009; Chen et al., 2011; Lv et al., 2016b), regulating transcription factors levels (Yu et al., 2015; Lv et al., 2016a) and modulating trichome numbers (Shi et al., 2017b; Yan et al., 2018). Semisynthesis of artemisinin by fermentation from engineered yeast is a feasible method of production of a precursor molecule, DHAA, which is easy to convert to artemisinin (Westfall et al., 2012).

Plant phytohormones increasing the secondary metabolites can be defined: the terpenoids, alkaloids and phenylpropanoids. In general, jasmonic acid (JA) is an important conserved elicitor of plant secondary metabolism. It triggers extensive transcriptional reprogramming and leads to an increase in the expression level of the gene encoding the key enzyme for secondary metabolism biosynthetic pathway (De Geyter et al., 2012). JA can induce the mRNA levels of *ORCA3* in *Catharanthus roseus*, which is an important transcription factor for the regulation of terpenoid indole alkaloid biosynthesis (Zhang et al., 2011). *ERF189* can be induced by JA in tobacco and it regulates the nicotine biosynthesis by directly binding to the GCC-box in the promoter region of putrescine *N*-methyltransferase (PMT) (Shoji et al., 2010). JA is involved in the biosynthesis of artemisinin as well. Previously, JA increased the expression levels of transcription factor *AaWRKY1*, which enhanced artemisinin content by directly binding to the promoter of *ADS* (Ma et al., 2009). We have found that *AaNAC1* was induced by JA and played an important role in the modulation of artemisinin biosynthesis (Lv et al., 2016a). Many transcription factors can be induced by JA and many are involved in artemisinin biosyntheses, such as *AaERF1*/*AaERF2, AaORA*, and *AaMYC2.* The AP2/ERF transcription factors *AaERF1*/*AaERF2* and *AaORA* positively regulate the biosynthesis of artemisinin (Yu et al., 2012; Lu et al., 2013). The bHLH transcription factor *AaMYC2* is a positive regulator for artemisinin biosynthesis by binding to the G-box-like motifs within the promoters of the artemisinin biosynthetic pathway gene *CYP71AV1* and *DBR2* (Shen et al., 2016).

*SQUAMOSA Promoter-Binding Protein-Like (SPL)* genes are functionally diverse, including modulation of grain size, grain shape, branching, vegetative phase change, shoot regeneration and flowering time (Wang et al., 2009; Jiao et al., 2010; Zhang et al., 2015; Si et al., 2016). Recently, some studies indicate that *SPL* transcription factors can regulate secondary metabolism biosynthesis. AtSPL9 can regulate anthocyanin accumulation by inhibiting expression levels of anthocyanin biosynthetic pathway genes through destabilization of the Myb-bHLH-TTG1 complex (Gou et al., 2011). At the same time, AtSPL9 can regulate the content of (E)-β-caryophyllene in *Arabidopsis thaliana* by binding to the promoter of *TPS21* (Yu et al., 2015). PpSPL1 negatively regulates the anthocyanin level in the peach flesh by inhibiting the transactivation activity of *NAC* transcription factors *BL* and *PpNAC1* through destabilization of the Myb-bHLH-TTG1 complex (Zhou et al., 2015).

Here, we also provide evidence that SPL transcription factors can regulate the artemisinin content by binding to the promoter of artemisinin biosynthesis gene *DBR2*. We provide evidence that overexpression of *AaSPL2* enhances artemisinin content, indicating that *AaSPL2* is a positive transcription factor of artemisinin biosynthesis in *A. annua.*

## Materials and Methods

### Plant Material

Seeds of “Huhao 1” (Shen et al., 2016), which is a high artemisinin yielding *A. annua* strain, were first surface-sterilized in 75% ethanol for 2 min, followed by 10% (v/v) NaOCl (sodium hypochlorite) solution for 10 min, and rinsed three times with sterile water. Subsequently, the seeds were planted on MS medium (Murashige and Skoog, 1962) with 3.0% sucrose (m/v) and 0.8% agar (m/v) and cultured with a photoperiod of 16 h light/8 h dark and light at 8,000 Lux at 28°C. Three weeks later, the leaves of young seedlings of *A. annua* (about 5–7 cm in height) were cut and used as explants for transformation with the *Agrobacterium tumefaciens*-mediated leaf disk transformation method (Zhang et al., 2009).

Seeds of tobacco (*Nicotiana benthamiana*) were sown directly on soil (vermiculite: perlite: peat moss = 7: 0.5: 2), 1 week later, the tobacco seedlings were transferred into the new pots and cultured with the same conditions as *A. annua*. Five weeks later, the leaves of tobacco can be used for *A. tumefaciens* infiltration assay.

### Construction and Transformation of *A. annua*

The ORF of AaSPL2 was introduced into the BamHI and SacI sites of pCAMBIA2300 (CAMBIA, Canberra, Australia) under the driven of CaMV35S promoter to generate CaMV35S:: AaSPL2 (overexpression vector). For RNAi interference assay, a 300 bp AaSPL2 fragment was fused into PENTR vector via the BP reaction (Invitrogen, United States) and then introduce into the pHELLSGATE12 vector via the LR reaction (Invitrogen, United States) to generate the final RNAi- AaSPL2 vector.

The overexpression constructs OX-*AaSPL2* and RNAi constructs *AaSPL2*-RNAi were introduced into *A. tumefaciens* strain EHA105 by a conventional freezing-and-melting method (Zhang et al., 2009). The method of transformation of *A. annua* was according to the previous description (Lv et al., 2016b).

### Plant Hormone Treatments

MJ was purchased from Sigma-Aldrich (St Louis, MO, United States). For plant hormone treatment, 4-week-old *A. annua* seedlings were treated with 100 μM MJ (Maes et al., 2011; Yu et al., 2012; Shen et al., 2016). Then the treated plants were sampled at time point 0, 1, 3, 6, 9, 12, and 24 h for RNA extracting and Q-PCR analysis.

### Isolation of Genomic DNA and PCR Analysis

The cetyltrimethylammonium bromide (CTAB) method was employed to obtain the genomic DNA of the transgenic plants (Allen et al., 2006). The forward primers of AaTGA6 were used to detect the transgenic plants. A 20 bp sequence of the vector was designed as reverse primers. The 20 ml PCR with 10 pmol forward primer, 10 pmol reverse primer, 50 ng of plant genomic DNA, 7 ml of sterilized water and 10 ml of rTaq DNA polymerase mix (TaKaRa, Japan) was performed. The amplification procedure consisted of a 5-min incubation at 95°C, followed by 30 cycles of 95°C for 30 s, and 60 s at 55°C for primer annealing and 72°C for 600 s elongation.

### RNA Isolation and Real-Time PCR Analysis

Young leaves of *A. annua* plants were used for RNA isolation by plant RNA Kit (Tiangen) according to the manufacturer’s instructions. The isolated total RNA was reverse-transcribed into cDNA using the TaKaRa cDNA synthesis kit. qRT-PCR assays using SYBR Green PCR Master Mix kit (Tiangen) as a detector were performed in Roch LightCycler^®^96 system according to the manufacturer’s instructions. The comparative cycle threshold (CT) method was estimated relative transcript levels of samples and means of triplicate independent PCRs. The *β-actin* gene of *A. annua* was used to estimate relative mRNA levels. The qRT-PCR primers are listed in Supplementary Table S1. The process for amplification was 5 min at 95°C, followed by 40 cycles of 95°C for 10 s, and 40 s at 60°C for primer annealing and elongation. Then a dissociation stage for 20 s at 95°C, 40 s at 60°C, and 20 s at 95°C.

The 2^-ΔΔCT^ method was used for estimating the relative gene expression level according to the following formula (Livak and Schmittgen, 2001):

$$\Delta \Delta \mathrm{CT}={\left(C_{T,\mathrm{Target}}-C_{T,\mathrm{actin}}\right)}_{\mathrm{Time}\times }-{\left(C_{T,\mathrm{Target}}-C_{T,\mathrm{actin}}\right)}_{Time0}$$

### Yeast One-Hybrid Assay

To analyze the specific binding of *AaSPL2* to GTAC-box of the *DBR2* promoters, the full length of *AaSPL2* was fused into the *Eco*RI–*Xho*I sites of the activation domain of the vector pB42AD (Clontech). Equal volume of the GTAC-box forward primer (10 μM) and reverse primer (10 μM) (Table 1) were mixed and incubated at these conditions: 95°C for 1 min, 85°C for 1 min, 75°C for 1 min, 65°C for 1 min, 55°C for 1 min, 45°C for 1 min, 35°C for 1 min, 25°C for 1 min. The reaction primer products were inserted into P178 vector (gifted by Dr. Yang Hongquan of Shanghai Jiao Tong University, Shanghai, China) with a single restriction site XhoI to form the reporter vector. Vectors containing the transcription factor gene and 3 × GTAC-box were introduced into the yeast strain EGY48 by LiAc mediated transformation method (Gietz and Schiestl, 2007). Yeast cells were selected on the synthetic dropout (SD) base medium with deficient in Trp and Ura for 3 days at 30°C. Then yeast cells were resuspended with sterile water and dropped on an 5-bromo-4-chloro-3-indolyl-beta-Dgalactopyrano-side (X-gal) medium plate (Zhang et al., 2015). The sequences of the 3 × GTAC-box fragment are listed in Supplementary Table S1.

**Table 1:** The character of SPLs in *A. annua*.

Gene name	Accession number	Gene length	PI	MW	miR156 target site
AaSPL1	MH090670	2484	4.9	206.73	
AaSPL2	MH090671	954	5.09	79.08	
AaSPL3	MH090672	2379	4.92	195.68	
AaSPL4	MH090673	879	5.1	72.71	✓
AaSPL5	MH090674	423	5.3	33.52	✓
AaSPL6	MH090675	2865	4.89	234.37	
AaSPL7	MH090676	1131	5.09	93.74	✓
AaSPL8	MH090677	1167	5.04	97.21	✓
AaSPL9	MH090678	906	5.1	74.37	✓
AaSPL10	MH090679	1284	5.02	107.01	✓
AaSPL11	MH090680	447	5.07	35.32	
AaSPL12	MH090681	516	5.27	40.57	✓
AaSPL13	MH090682	1197	5.03	99.08	✓
AaSPL14	MH090683	555	5.23	44.87	✓

### Dual-LUC Assay

For the dual-LUC assay, the promoter of *DBR2* was fused to firefly luciferase gene on the plasmid pGreenII 0800-LUC. The sequenced plasmid was introduced into *A. tumefaciens* strain EHA105. Then incubated Agrobacterium cells were harvested by centrifugation and resuspended in MS medium (containing 10 mM MES and 150 mM acetosyringone) to an OD600 nm of 0.6. After 3 h incubation at room temperature, bacteria (reporter strain DBR2pro::LUC mixed with effector strain 35Spro::*AaSPL2* 1:1) were injected into tobacco leaves. Forty-eight hours’ later, the injected tobacco leaves were collected for dual-LUC assay. The dual-Luciferase Reporter Assay system of Promega was used for dual-Luc assays according to the manufacturer’s instructions.

### Quantification of Artemisinin Using HPLC-ELSD (HPLC-Evaporative Light Scattering Detection)

The upper parts leaves of *A. annua* were collected at pre-flowering stage and dried at 50°C for 24 h. then all the leaves were ground into powder. A sample of 0.1 g powder was suspended in ethanol (2 mL) for ultrasonication. Sampling was carried out in triplicate. Thirty min later, Samples were centrifuged for 15 min, 4°C, at 12 000 g. The supernatant was filtered through a 0.22 mm pore size filter and then analyzed by the Waters Alliance 2695 HPLC system. Artemisinin standard (98% pure) was purchased from Sigma. The approach for detecting artemisinin using HPLC-ELSD has already been described (Zhang et al., 2009).

## Results

### Characterization of AaSPLs in *A. annua*

The SQUAMOSA promoter-binding protein (SBP) genes contain a conserved domain, which encodes 76 amino acid residues in length for the interaction with DNA (Cardon et al., 1999). To identify the SPLs in *A. annua*, the putative conserved SBP domain was used for searching the transcriptome database (Graham et al., 2010; Hao et al., 2017) using the HMMER3.0 search program^1^ (Finn et al., 2011). Through molecular cloning, we identified a total of 14 SPLs (*AaSPL1*∼*AaSPL14*) in *A. annua* with the GenBank accession number (Table 1). The open reading frame (ORF) of the SPL is varied in length from 447bp to 2865bp. All the SPLs encode protein products 149 to 955 amino acids with the calculated isoelectric point^2^ of 4.89 to 5.3. The details of the gene character of SPLs are shown in the Table 1.

MiR156 plays important role in modulation of transcription levels of targeting SPLs for translational repression or cleavage (Wang et al., 2009; Wu et al., 2009). So the miR156 in *A. annua* has been studied recently (Pani et al., 2011). To confirm the targets of miR156, we screened all of the SPLs in *A. annua.* Nine of SPLs have target site of miR156 and they are predicted to be the potential targets of miR156 (Table 1).

### Expression Patterns of SPLs in *A. annua*

**FIGURE 2:**
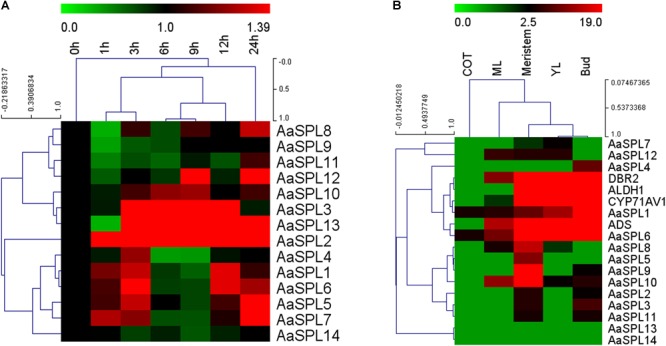
Heat map representation of expression profiles of SPL transcription factors. **(A)** The heat map represents the SPL transcription factors expression by Q-PCR under the treatment of JA at time point of 0,1,3,6,9,12, and 24 h. **(B)** Expression data for SPL genes, *ADS, CYP71AV1, DBR2*, and *ALDH1* in cotyledon, young leaves, mature leaves, buds and meristems. Expression data were detected by Q-PCR and the results were visualized using Mev 4.9 software. β-actin was used as a reference. Red indicates high expression; green, low expression.

JA plays a crucial role in inducing the artemisinin accumulation (Jing et al., 2009; Maes et al., 2011; Yu et al., 2012). We hypothesized that JA may be used for screening the SPL which takes part in modulating artemisinin biosynthesis in *A. annua*. First, we checked mRNA levels of AaSPL1∼ AaSPL14 at different time points (0, 1, 3, 6, 9, 12, and 24 h) after treatment with JA (100 μM). To this end, the expression intensity and hierarchical clustering (HCL) of SPL genes were formatted as a heatmap by Mev4.9.0 software.^3^ Exogenous application of JA to *A. annua* seedlings resulted in strong activation of both AaSPL2 and AaSPL13 (Figure 2A), indicating that both of them may play a role in the regulation of artemisinin.

In the process of artemisinin biosynthesis, the artemisinin biosynthetic pathway genes *ADS, CYP71AV1, DBR2*, and *ALDH1* mRNA levels were accumulated in the young leave tissues (YL) and buds, confirming previous studies that artemisinin was produced in the glandular trichomes (Maes et al., 2011). To confirm the expression files of SPLs of *A. annua*, we tested SPL expression levels in the RNA-sequence of different tissues (Graham et al., 2010). As shown in Figure 2B, heatmap results indicated that the mRNA levels of *AaSPL2* are higher in bud compared with AaSPL13, therefore, we focus our attention on *AaSPL2* according to the results of the expression profiles of SPLs.

### Characterization of *AaSPL2*

**FIGURE 3:**
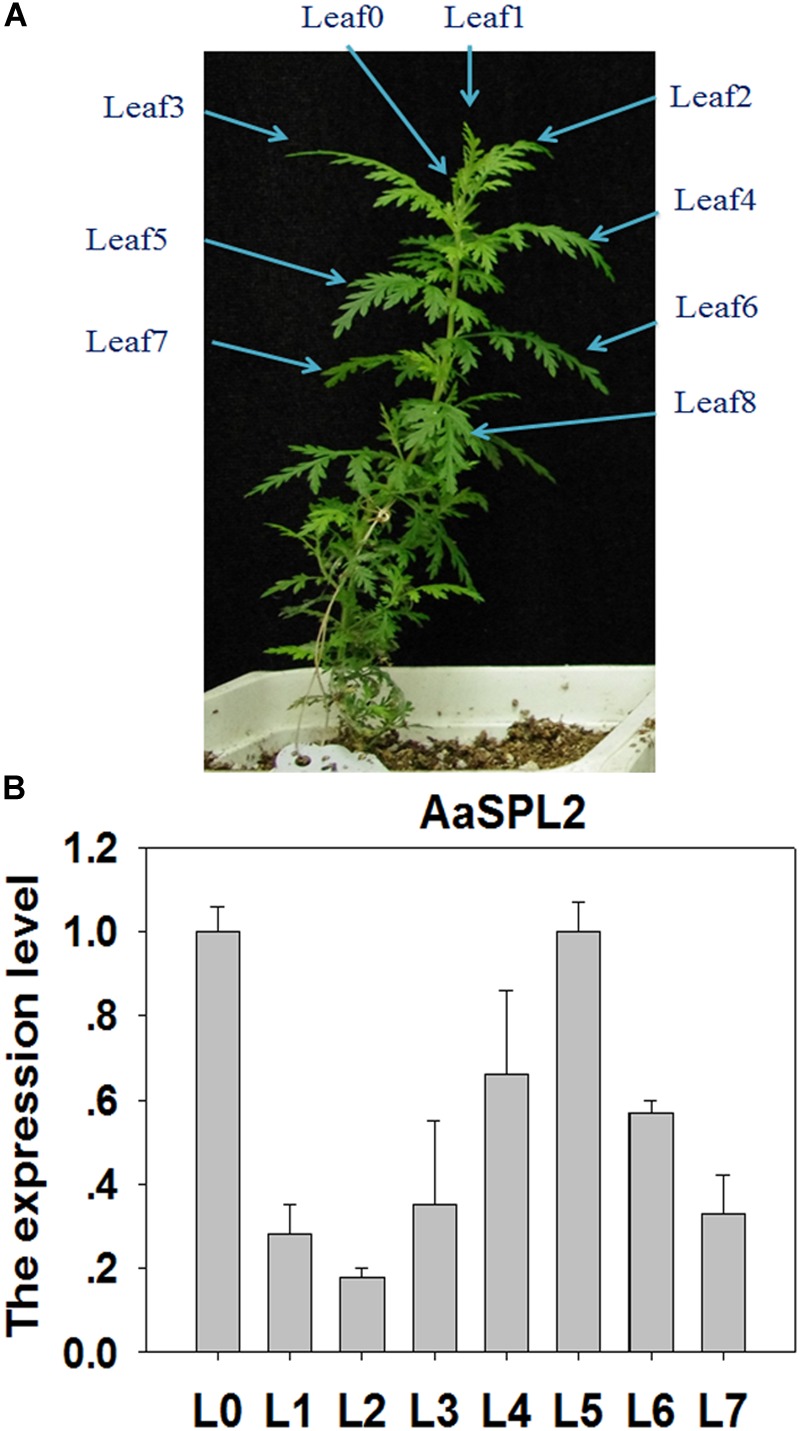
Expression profiles of *AaSPL2* in the different leaves. **(A)** L0 to L7 were labeled on the 6-week-old *A. annua* seedling. **(B)** Expression of *AaSPL2* in the different leaves. L0 to L7 means leaves 0 to leaves 7. The expression level is normalized by Leaf0 value. Bars represent means ± SE (*n* = 3).

SPL transcription factors are known to regulate the biosynthesis of secondary metabolites, such as sesquiterpene, flavonoid (Gou et al., 2011; Yu et al., 2015). We hypothesized that SPL may modulate the biosynthesis of sesquiterpene lactone artemisinin by directly or indirectly regulation of artemisinin biosynthetic pathway genes. To investigate the gene function and control mechanism, a 954-bp segment of the *AaSPL2* ORF region was isolated from cDNA. The full-length cDNA of AaSPL2 encodes a polypeptide of 318 amino acid residues with a calculated mass of 79 kDa and a pI of 5.09 (Table 1). We also detected the gene expression level of *AaSPL2* in leaf0∼leaf7 (L0∼L7) (Figure 3A). The Q-PCR results indicated that *AaSPL2* expressed at a high level in the L0, L4, L5, and L6 (Figure 3B).

### Phylogenetic Analysis of *AaSPL2* Proteins

**FIGURE 4:**
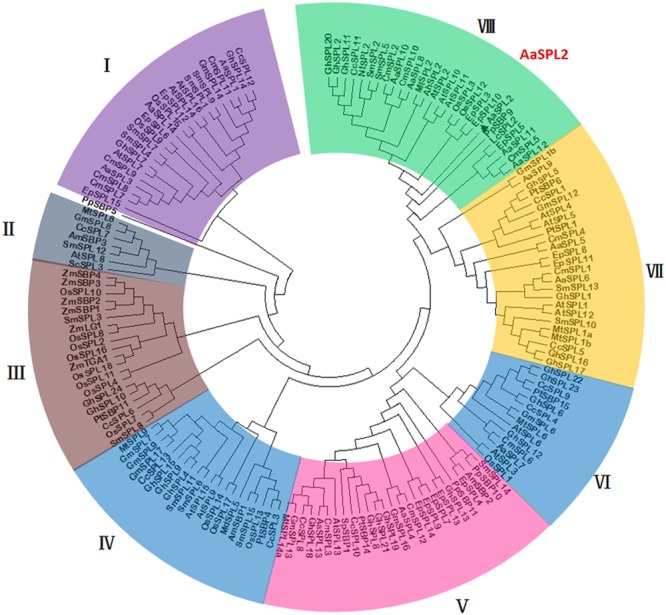
Phylogenetic tree of SPL transcription factors from selected plant species. The tree was constructed using MEGA 7 software with Neighbor–Joining method: bootstrap analysis of 1,000 replicates. The numbers at the nodes indicate the bootstrap values. The bar at the bottom indicates the relative divergence of the sequences examined. *AaSPL2* was marked with a black triangle.

The analysis on full-length nucleotide sequence showed that *AaSPL2* contains a conserved SBP domain in the 289–471 regions. A phylogenetic tree by using nucleic acid sequences was built to investigate the phylogenetic relationships among the SPL proteins in *A. annua*, Arabidopsis and rice other species. The results indicated that *AaSPL2* belongs to the subfamily of AtSPL2/10/11 transcription factors (Figure 4). AtSPL2/10/11 has been reported to modulate vegetative phase change and trichome development (Shikata et al., 2009), suggesting that *AaSPL2* has a potential role in the regulation of phase transition or trichome initiation in *A. annua.*

### Subcellular Localization of AaSPL2

**FIGURE 5:**
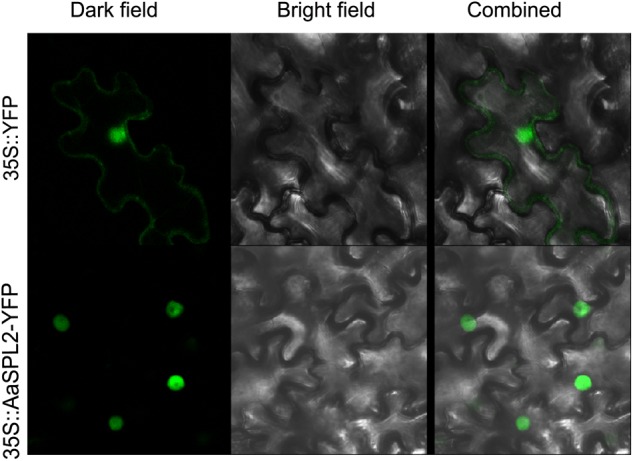
Subcellular localizations of AaSPL2 in *N. benthamiana* leaves. Constructs 35S::AaSPL2–YFP were introduced into *Agrobacterium* and transferred into *N. benthamian* by infiltration. Constructs 35S::YFP were used as control.

To investigate the subcellular localization of the AaSPL2 protein, a YFP was fused to the C-terminus of AaSPL2 and the AaSPL2-YFP construct was expressed under the control of the double 35S Cauliflower mosaic virus (CaMV) promoter. Agroinfiltration method was performed to infiltrate *N. tabacum* leaves (Zhang et al., 2015). Controls showed GFP expressed throughout the cytoplasm (Figure 5), whereas the AaSPL2-YFP fusion was targeted to the nucleus (Figure 5).

### AaSPL2 Activates the Transcription of *DBR2 in vivo*

**FIGURE 6:**
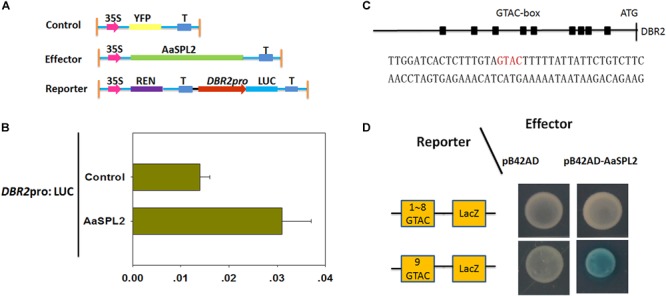
AaSPL2 promotes transactivation of *DBR2*. **(A)** A Schematic representation of control, effectors and reporters used in the dual-LUC assays is shown. The LUC reporter constructs harboring *DBR2* promoters were used as reporter (*DBR2*pro: LUC). **(B)** Effector constructs harboring the AaSPL2 full length were driven by the CaMV35S promoter. The LUC activities were normalized by Renilla (REN) luciferase activities. The YFP driven by 35S promoter was used as negative control. **(C)** A Schematic representation of the “GTAC” elements in the *DBR2* promoters. Black box indicates the “GTAC” elements in the *DBR2* promoters. The DNA sequences used in the Y1H assays is shown. **(D)** Yeast one-hybrid assay indicates AaSPL2 binding to the promoter of *DBR2.* A Schematic in the left representation of the reporter constructs was used in the yeast one-hybrid assay. Right panel represents the yeast cells. Yeast strains were diluted with sterile water and 6 μL of each dilution was plated on synthetic dropout media without Trp and Ura (SD-T-U) plate plus X-gal.

The SQUAMOSA promoter binding protein transcription factors can activate the transcription of MADS transcription factors by binding to “GTAC” *cis*-element in the promoter (Franco-Zorrilla et al., 2014). There are 9 “GTAC” *cis*-elements in the promoter of *DBR2* (Jiang et al., 2014). Thus, we hypothesized that *AaSPL2* may potentially induce the transcription of *DBR2.* First, a dual luciferase assay (dual-LUC) was employed to detect if the expression of *DBR2* can be regulated by the product of the transcription factor *AaSPL2* (Figure 6A). The results of dual-LUC indicated that *AaSPL2* activates the transcription of *DBR2* (Figure 6B).

To further substantiate this finding, we tested whether *AaSPL2* is able to bind to the “GTAC” box in the *DBR2* promoters. Therefore, the ninth “GTAC” elements were cloned into the reporter vector p178 (Figure 6C). The results of the Y1H indicated that *AaSPL2* can bind to the “GTAC” of the *DBR2* promoters directly (Figure 6D).

### Overexpression of *AaSPL2* in *A. annua* Increases Artemisinin Biosynthesis

**FIGURE 7:**
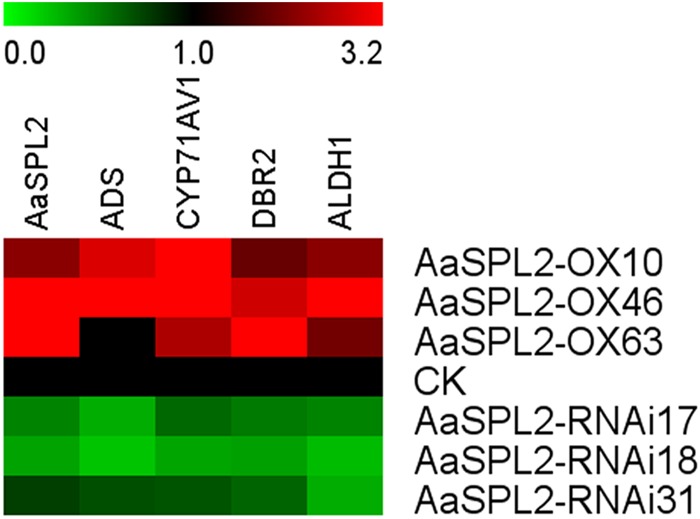
Heat map showing the expression levels of AaSPL2 and artemisinin biosynthesis pathway genes. Expression data were detected by Q-PCR and the results were visualized using Mev 4.9 software. *β-actin* was used as a reference. Red indicates high expression; green, low expression.

**FIGURE 8:**
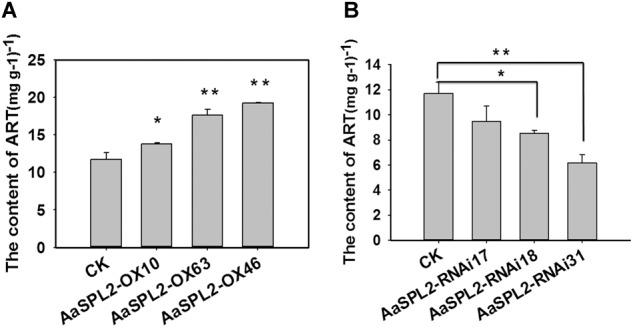
Artemisinin content in the transgenic plants was determined by HPLC. **(A)** Artemisinin content was detected in the *AaSPL2* overexpression plants. **(B)** Artemisinin content was detected in the AaSPL2 RNAi plants. Error bars indicate SD (*n* = 3). ^∗^*p* < 0.05, ^∗∗^*p* < 0.01.

The function of SPL transcription factors is diverse. To investigate the functional role of *AaSPL2*, we overexpressed *AaSPL2* (abbreviated as *AaSPL2*-OX) in *A. annua*. The transgenic plants were detected by PCR. Overexpression of *AaSPL2* in *A. annua* resulted in enhanced *AaSPL2* expression upon wild-type (Figure 7). The expression levels of artemisinin biosynthetic pathway genes *ADS, CYP71AV1, DBR2*, and *ALDH1* were also detected. Similar results were obtained with the expression levels of *ADS, CYP71AV1, DBR2*, and *ALDH1* (Figure 7). Therefore, to investigate whether *AaSPL2* is a positive regulator for artemisinin biosynthesis, the artemisinin content was detected. In the *AaSPL2* overexpression plants, the artemisinin and DHAA content was increased to 33–86% and 26–159%, respectively (Figure 8A and Supplementary Figure S1A). In the *AaSPL2*-RNAi transgenic plants, the mRNA levels of *AaSPL2* and artemisinin biosynthetic pathway genes all decreased (Figure 7). The artemisinin and DHAA content was decreased by 33–65% and 3–16%, respectively (Figure 8B and Supplementary Figure S1B). Thus, *AaSPL2* is a positive regulator for artemisinin biosynthesis. The transgenic plants displaying normal phenotype overexpressed in Arabidopsis (Supplementary Figure S2) and in *A. annua* (Supplementary Figure S3).

## Discussion

### MJ Induced Artemisinin Biosynthesis

Since JA and its cyclic precursors and derivatives play important roles in inducing the genes encoding enzymes involving in the biosynthesis of secondary metabolites in the plant (Van der Fits and Memelink, 2000; De Boer et al., 2011). So JA becomes the hotspot as the secondary metabolites inducer. Previously, it was shown that overexpression of artemisinin biosynthetic pathway genes (*ADS, CYP71AV1, DBR2, ALDH1, CPR*) can redirect carbon flux to the artemisinin pathway and increase artemisinin contents for several folds (Chen et al., 2013; Shi et al., 2017a). JA enhances artemisinin biosynthetic pathway genes mRNA levels and plays the same roles as overexpression artemisinin biosynthetic pathway genes (Maes et al., 2011). JA improved artemisinin content by increasing mRNA levels of transcription factors as well, which regulate gene expression level through specific binding to *cis*-acting elements in the target genes promoter. Many transcription factors are involved in JA signaling cascades and regulate the biosynthesis of artemisinin. Such as AP2/ERF transcription factors *AaERF1* and *AaERF2*, they bind to the promoter of *ADS* and *CYP71AV1* directly (Yu et al., 2012). Another AP2/ERF transcription factor AaORA (Lu et al., 2013), which increases artemisinin content by improving artemisinin biosynthetic pathway genes mRNA levels. AaMYC2 and AaNAC1 activate the expression level of artemisinin biosynthesis pathway genes *CYP71AV1, DBR2*, and *ADS*, respectively (Lv et al., 2016a; Shen et al., 2016). *AaSPL2* mRNA level was enhanced under the treatment of JA and the content of artemisinin was increased in the *AaSPL2* overexpression plants also (Figure 2). AtSPL9 attended the JA pathway through interacting with known JA signaling factor (such as JAZ3) to regulate biosynthesis of secondary metabolism glucosinolate and β-caryophyllene (Yu et al., 2015; Mao et al., 2017). On the other hand, JA improved artemisinin content by increasing of trichome number (Maes et al., 2011). Artemisinin was specifically synthesized in the glandular trichome and was related to the trichome number. The increase of trichome number may decrease feedback Inhibition of artemisinin content (Arsenault et al., 2010). In our study, trichome number changes not obviously (Supplementary Figure S3F), suggesting that AaSPL2 may not take part in trichome regulation.

### SPL Transcription Factors Regulation of Terpenoid Products

Through genome-wide identification, a total of 14 SPLs have been found in *Salvia miltiorrhiza* (Zhang et al., 2014). There are 19 SPL genes in the rice and 16 SPLs in Arabidopsis (Birkenbihl et al., 2005; Manning et al., 2006). A systematical study reported that 59 SPLs were identified in the Genome-wide of *G. hirsutum*. SPLs play important roles in fiber initiation, branching, root growth, grain size and yield, suggesting that SPL transcription factors play key roles in *G. hirsutum* growth and development. The reasons for so many SPLs in the *G. hirsutum* are the tandem duplication events or by assembly error (Cai et al., 2018). In this study, a total of 14 SPLs were cloned. AaSPL2 mainly expressed in the buds (Figure 2B) and induced by MJ (Figure 2A), play important role in artemisinin biosynthesis (Figure 8). However, AaSPL3 has the same expression patterns with AaSPL2 (Figure 2A,B), therefore, it is a potential gene for improving artemisinin content.

SPL transcription factors can regulate gene expression through binding to *cis*-regulatory specific core “GTAC” consensus sequences in the promoters of their target genes. Previously, the core “GTAC” consensus sequence of *DFR* has been identified as the binding motif of SPL9 (Gou et al., 2011). Plant terpenoids have important physiological and ecological functions, such as serving as signals in biocommunications and interactions, and phytoalexins in defense against pests and pathogens (Sharma et al., 2017). Terpene synthase 21 (TPS21) is responsible for the vast majority of floral sesquiterpenes and it can catalyze the conversion of FPP into β-caryophyllene. Transcription factor SPL9 directly binds to *TPS21* promoters and activates its expression to regulate the biosynthesis of β-caryophyllene (Yu et al., 2015). Artemisinin is a sesquiterpene, hence, we postulated that SPL in *A. annua* may function similarly as an *SPL* in Arabidopsis. The phylogenetic tree indicated that AaSPL2 may homologous to the AtSPL3 in Arabidopsis.

The expression patterns indicated that *AaSPL2* mainly expressed in the buds (Figure 2), which is partly same as artemisinin biosynthetic genes expression patterns, so *AaSPL2* in *A. annua* may play the role in the regulation of sesquiterpene artemisinin. *AaSPL2*-OX and *AaSPL2-*RNAi results show that SPL2 can modulate artemisinin (Figure 8) and DHAA accumulation (Supplementary Figure S1). The fact that the overexpression of *AaSPL2* partially increases the transcription levels of artemisinin biosynthetic gene *ADS, CYP71AV1, DBR2*, and *ALDH1* supported the hypothesis that enhancing of *AaSPL2* expression level may be a plausible mechanism for increase artemisinin content.

## Author Contributions

ZL, KT, WC, and LZ conceived and designed the entire research plans. BP, YL, and ZL performed the most of the experiments. BP and ZL provided the technical assistance. ZL wrote the manuscript. ZL, YW, and LZ helped with the organization and editing.

## Conflict of Interest Statement

The authors declare that the research was conducted in the absence of any commercial or financial relationships that could be construed as a potential conflict of interest.

## Footnotes

1 http://hmmer.janelia.org

2 https://web.expasy.org/compute_pi/

3 http://www.mybiosoftware.com/mev-4-6-2-multiple-experiment-viewer.html

## Supplementary Material

FIGURE S1 DHAA content in the transgenic plants was determined by HPLC. **(A)** DHAA content was detected in the *AaSPL2* overexpression plants. **(B)** DHAA content was detected in the AaSPL2 RNAi plants. Error bars indicate SD (*n* = 3). ^∗∗^*p* < 0.01.

FIGURE S2 The phenotype of AaSPL2 overexpressed in Arabidopsis.

FIGURE S3 The phenotype of AaSPL2 overexpressed in *A. annua*. **(A)** A schematic representation of overexpression vector and RNAi vector used in the *A. annua* transformation is shown. **(B)** the phenotype of overexpression and RNAi transgenic plants. **(C)** Glandular trichome of control. **(D)** Glandular trichome of overexpression plants. **(E)** Glandular trichome of RNAi plants. **(F)** Trichome number of *A. annua.* Scale bar represents 0.5 mm.

TABLE S1 Primers used in this study.

## Abbreviations

ADS: amorpha-4,11-diene synthase
ALDH1: aldehyde dehydrogenase 1
CYP71AV1: amorphadiene 12-hydroxylase
DBR2: artemisinic aldehyde Δ11(13) reductase
DHAA: dihydroartemisinic acid
YFP: yellow fluorescent protein

## References

[B1] Allen G. C. Flores-Vergara M. A. Krasynanski S. Kumar S. Thompson W. F. (2006). A modified protocol for rapid DNA isolation from plant tissues using cetyltrimethylammonium bromide. *Nat. Protoc.* 1 2320–2325. 10.1038/nprot.2006.384 17406474

[B2] Arsenault P. R. Vail D. Wobbe K. K. Erickson K. Weathers P. J. (2010). Reproductive development modulates gene expression and metabolite levels with possible feedback inhibition of artemisinin in *Artemisia annua*. *Plant Physiol.* 154 958–968. 10.1104/pp.110.162552 20724645 PMC2949044

[B3] Birkenbihl R. P. Jach G. Saedler H. Huijser P. (2005). Functional dissection of the plant-specific SBP-domain, overlap of the DNA-binding and nuclear localization domains. *J. Mol. Biol.* 352 585–596. 10.1016/j.jmb.2005.07.013 16095614

[B4] Cai C. Guo W. Zhang B. (2018). Genome-wide identification and characterization of SPL transcription factor family and their evolution and expression profiling analysis in cotton. *Sci. Rep.* 8:762. 10.1038/s41598-017-18673-4 29335584 PMC5768680

[B5] Cardon G. Höhmann S. Klein J. Nettesheim K. Saedler H. Huijser P. (1999). Molecular characterisation of the *Arabidopsis* SBP-box genes. *Gene* 237 91–104. 10.1016/S0378-1119(99)00308-X 10524240

[B6] Chen J. L. Fang H. M. Ji Y. P. Pu G. B. Guo Y. W. Huang L. L. (2011). Artemisinin biosynthesis enhancement in transgenic *Artemisia annua* plants by downregulation of the (-caryophyllene synthase dene. *Planta Med.* 77 1759–1765. 10.1055/s-0030-1271038 21509717

[B7] Chen Y. Shen Q. Wang Y. Wang T. Wu S. Zhang L. (2013). The stacked over-expression of *FPS, CYP71AV1* and *CPR* genes leads to the increase of artemisinin level in *Artemisia annua* L. *Plant Biotechnol. Rep.* 7 287–295. 10.1007/s11816-012-0262-z

[B8] De Boer K. Tilleman S. Pauwels L. Vanden B. R. De Sutter V. Vanderhaeghen R. (2011). APETALA2/ETHYLENE RESPONSE FACTOR and basic helix-loop-helix tobacco transcription factors cooperatively mediate jasmonate-elicited nicotine biosynthesis. *Plant J.* 66 1053–1065. 10.1111/j.1365-313X.2011.04566.x 21418355

[B9] De Geyter N. Gholami A. Goormachtig S. Goossens A. (2012). Transcriptional machineries in jasmonate-elicited plant secondary metabolism. *Trends Plant Sci.* 17 349–359. 10.1016/j.tplants.2012.03.001 22459758

[B10] Duke S. O. Paul R. N. (1993). Development and fine structure of the glandular trichomes of *Artemisia annua* L. *Int. J. Plant Sci.* 154 107–118. 10.2307/2995610

[B11] Finn R. D. Clements J. Eddy S. R. (2011). HMMER web server, interactive sequence similarity searching. *Nucleic Acids Res.* 39 W29–W37. 10.1093/nar/gkr367 21593126 PMC3125773

[B12] Franco-Zorrilla J. M. López-Vidriero I. Carrasco J. L. Godoy M. Vera P. Solano R. (2014). DNA-binding specificities of plant transcription factors and their potential to define target genes. *Proc. Natl. Acad. Sci. U.S.A.* 111 2367–2372. 10.1073/pnas.1316278111 24477691 PMC3926073

[B13] Gietz R. D. Schiestl R. H. (2007). Large-scale high-efficiency yeast transformation using the LiAc/SS carrier DNA/PEG method. *Nat. Protoc.* 2 38–41. 10.1038/nprot.2007.15 17401336

[B14] Gou J. Y. Felippes F. F. Liu C. J. Weigel D. Wang J. W. (2011). Negative regulation of anthocyanin biosynthesis in *Arabidopsis* by a miR156-targeted SPL transcription factor. *Plant Cell* 23 1512–1522. 10.1105/tpc.111.084525 21487097 PMC3101539

[B15] Graham I. A. Besser K. Blumer S. Branigan C. A. Czechowski T. Elias L. (2010). The genetic map of *Artemisia annua* L. identifies loci affecting yield of the antimalarial drug artemisinin. *Science* 327 328–331. 10.1126/science.1182612 20075252

[B16] Hao X. Zhong Y. Fu X. Lv Z. Shen Q. Yan T. (2017). Transcriptome analysis of genes associated with the artemisinin biosynthesis by jasmonic acid treatment under the light in *Artemisia annua*. *Front. Plant Sci.* 8:971. 10.3389/fpls.2017.00971 28642777 PMC5463050

[B17] Jiang W. Lu X. Qiu B. Zhang F. Shen Q. Lv Z. (2014). Molecular cloning and characterization of a trichome-specific promoter of artemisinic aldehyde Δ11 (13). reductase (DBR2). in *Artemisia annua*. *Plant Mol. Biol. Rep.* 32 82–91. 10.1007/s11105-013-0603-2

[B18] Jiao Y. Wang Y. Xue D. Wang J. Yan M. Liu G. (2010). Regulation of *OsSPL14* by OsmiR156 defines ideal plant architecture in rice. *Nat. Genet.* 42 541–544. 10.1038/ng.591 20495565

[B19] Jing F. Zhang L. Li M. Tang Y. Wang Y. Wang Y. (2009). Abscisic acid (ABA). Treatment increases artemisinin content in *Artemisia annua* by enhancing the expression of genes in artemisinin biosynthetic pathway. *Biologia* 64 319–323. 10.2478/s11756-009-0040-8

[B20] Kindermans J. Pilloy J. Olliaro P. Gomes M. (2007). Ensuring sustained ACT production and reliable artemisinin supply. *Malar. J.* 6:125. 10.1186/1475-2875-6-125 17868471 PMC2014776

[B21] Livak K. J. Schmittgen T. D. (2001). Analysis of relative gene expression data using real-time quantitative PCR and the 2– ΔΔCT method. *Methods* 25 402–408. 10.1006/meth.2001.1262 11846609

[B22] Lu X. Zhang L. Zhang F. Jiang W. Shen Q. Zhang L. (2013). AaORA, a trichome-specific AP2/ERF transcription factor of *Artemisia annua*, is a positive regulator in the artemisinin biosynthetic pathway and in disease resistance to *Botrytis cinerea*. *New Phytol.* 198 1191–1202. 10.1111/nph.12207 23448426

[B23] Lv Z. Wang S. Zhang F. Chen L. Hao X. Pan Q. (2016a). Overexpression of a novel NAC domain-containing transcription factor (*AaNAC1*). Enhances the content of artemisinin and increases tolerance to drought and *Botrytis cinerea* in *Artemisia annua*. *Plant Cell Physiol.* 57 1961–1971. 10.1093/pcp/pcw118 27388340

[B24] Lv Z. Zhang F. Pan Q. Fu X. Jiang W. Shen Q. (2016b). Branch pathway blocking in *Artemisia annua* is a useful method for obtaining high yield Artemisinin. *Plant Cell Physiol.* 57 588–602. 10.1093/pcp/pcw014 26858285

[B25] Ma D. Pu G. Lei C. Ma L. Wang H. Guo Y. (2009). Isolation and characterization of *AaWRKY1*, an *Artemisia annua* transcription factor that regulates the amorpha-4, 11-diene synthase gene, a key gene of artemisinin biosynthesis. *Plant Cell Physiol.* 50 2146–2161. 10.1109/TPWRD.2004.829125 19880398

[B26] Maes L. Van Nieuwerburgh F. C. W. Zhang Y. Reed D. W. Pollier J. Vande Casteele S. R. F. (2011). Dissection of the phytohormonal regulation of trichome formation and biosynthesis of the antimalarial compound artemisinin in *Artemisia annua* plants. *New Phytol.* 189 176–189. 10.2307/40960883 20874804

[B27] Manning K. Tör M. Poole M. Hong Y. Thompson A. J. King G. J. (2006). A naturally occurring epigenetic mutation in a gene encoding an SBP-box transcription factor inhibits tomato fruit ripening. *Nat. Genet.* 38 948–952. 10.1038/ng1841 16832354

[B28] Mao Y. B. Liu Y. Q. Chen D. Y. Chen F. Y. Fang X. Hong G. J. (2017). Jasmonate response decay and defense metabolite accumulation contributes to age-regulated dynamics of plant insect resistance. *Nat. Commun.* 8:13925. 10.1038/ncomms13925 28067238 PMC5233801

[B29] Murashige T. Skoog F. (1962). A revised medium for rapid growth and bio assays with tobacco tissue cultures. *Physiol. Plant.* 15 473–497. 10.1111/j.1399-3054.1962.tb08052.x

[B30] Paddon C. J. Westfall P. J. Pitera D. J. Benjamin K. Fisher K. Mcphee D. (2013). High-level semi-synthetic production of the potent antimalarial artemisinin. *Nature* 496 528–532. 10.1038/nature12051 23575629

[B31] Pani A. Mahapatra R. K. Behera N. Naik P. K. (2011). Computational identification of sweet wormwood (*Artemisia annua*). microRNA and their mRNA targets. *Genomics Proteomics Bioinformatics* 9 200–210. 10.1016/S1672-0229(11)60023-5 22289476 PMC5054163

[B32] Peplow M. (2016). Synthetic biology’s first malaria drug meets market resistance. *Nature* 530 389–390. 10.1038/530390a 26911755

[B33] Sharma E. Anand G. Kapoor R. (2017). Terpenoids in plant and arbuscular mycorrhiza-reinforced defence against herbivorous insects. *Ann. Bot.* 119 791–801. 10.1093/aob/mcw263 28087662 PMC5378189

[B34] Shen Q. Lu X. Yan T. Fu X. Lv Z. Zhang F. (2016). The jasmonate-responsive *AaMYC2* transcription factor positively regulates artemisinin biosynthesis in *Artemisia annua*. *New Phytol.* 210 1269–1281. 10.1111/nph.13874 26864531

[B35] Shi P. Fu X. Liu M. Shen Q. Jiang W. Li L. (2017a). Promotion of artemisinin content in *Artemisia annua* by overexpression of multiple artemisinin biosynthetic pathway genes. *Plant Cell Tissue Organ Cult.* 129 251–259. 10.1007/s11240-017-1173-z

[B36] Shi P. Fu X. Shen Q. Liu M. Pan Q. Tang Y. (2017b). The roles of AaMIXTA1 in regulating the initiation of glandular trichomes and cuticle biosynthesis in *Artemisia annua*. *New Phytol.* 1 261–276. 10.1111/nph.14789 28940606

[B37] Shikata M. Koyama T. Mitsuda N. Ohme-Takagi M. (2009). Arabidopsis SBP-box genes SPL10, SPL11 and SPL2 control morphological change in association with shoot maturation in the reproductive phase. *Plant Cell Physiol.* 50 2133–2145. 10.1093/pcp/pcp148 19880401

[B38] Shoji T. Kajikawa M. Hashimoto T. (2010). Clustered transcription factor genes regulate nicotine biosynthesis in tobacco. *Plant Cell* 22 3390–3409. 10.1105/tpc.110.078543 20959558 PMC2990138

[B39] Si L. Chen J. Huang X. Gong H. Luo J. Hou Q. (2016). OsSPL13 controls grain size in cultivated rice. *Nat. Genet.* 48 447–456. 10.1038/ng.3518 26950093

[B40] Van der Fits L. Memelink J. (2000). ORCA3, a jasmonate-responsive transcriptional regulator of plant primary and secondary metabolism. *Science* 289 295–297. 10.1126/science.289.5477.295 10894776

[B41] Wang J. W. Czech B. Weigel D. (2009). miR156-regulated SPL transcription factors define an endogenous flowering pathway in *Arabidopsis thaliana*. *Cell* 138 738–749. 10.1016/j.cell.2009.06.014 19703399

[B42] Westfall P. J. Pitera D. J. Lenihan J. R. Eng D. Woolard F. X. Regentin R. (2012). Production of amorphadiene in yeast, and its conversion to dihydroartemisinic acid, precursor to the antimalarial agent artemisinin. *Proc. Natl. Acad. Sci. U.S.A.* 109 E111–E118. 10.1073/pnas.1110740109 22247290 PMC3271868

[B43] World Health Organization (2014). *World Malaria Report 2014.* Geneva: World Health Organization 10.30875/9f925144-en

[B44] Wu G. Park M. Y. Conway S. R. Wang J. W. Weigel D. Poethig R. S. (2009). The sequential action of miR156 and miR172 regulates developmental timing in *Arabidopsis*. *Cell* 138 750–759. 10.1016/j.cell.2009.06.031 19703400 PMC2732587

[B45] Yan T. Li L. Xie L. Chen M. Shen Q. Pan Q. (2018). A novel HD-ZIP IV/MIXTA complex promotes glandular trichome initiation and cuticle development in *Artemisia annua*. *New Phytol.* 2 567–578. 10.1111/nph.15005 29377155

[B46] Yu Z. Wang L. Zhao B. Shan C. Zhang Y. Chen D. (2015). Progressive regulation of sesquiterpene biosynthesis in *Arabidopsis* and patchouli (*Pogostemon cablin*). by the miR156-targeted SPL transcription factors. *Mol. Plant* 8 98–110. 10.1093/mp/ssu127 25578275

[B47] Yu Z. X. Li J. X. Yang C. Q. Hu W. L. Wang L. J. Chen X. Y. (2012). The jasmonate-responsive AP2/ERF transcription factors *AaERF1* and AaERF2 positively regulate artemisinin biosynthesis in *Artemisia annua* L. *Mol. Plant* 5 356–365. 10.1093/mp/ssr087 22104293

[B48] Zhang H. Hedhili S. Montiel G. Zhang Y. Chatel G. Pré M. (2011). The basic helix-loop-helix transcription factor CrMYC2 controls the jasmonate-responsive expression of the ORCA genes that regulate alkaloid biosynthesis in *Catharanthus roseus*. *Plant J.* 67 61–71. 10.1111/j.1365-313X.2011.04575.x 21401746

[B49] Zhang L. Jing F. Li F. Li M. Wang Y. Wang G. (2009). Development of transgenic *Artemisia annua* (Chinese wormwood). Plants with an enhanced content of artemisinin, an effective anti-malarial drug, by hairpin-RNA-mediated gene silencing. *Biotechnol. Appl. Biochem.* 52 199–207. 10.1042/BA20080068 18564056

[B50] Zhang L. Wu B. Zhao D. Li C. Shao F. Lu S. (2014). Genome-wide analysis and molecular dissection of the SPL gene family in *Salvia miltiorrhiza*. *J. Integr. Plant Biol.* 56 38–50. 10.1111/jipb.12111 24112769

[B51] Zhang T. Lian H. Tang H. Dolezal K. Zhou C. Yu S. (2015). An intrinsic microRNA timer regulates progressive decline in shoot regenerative capacity in plants. *Plant Cell* 27 349–360. 10.1105/tpc.114.135186 25649435 PMC4456919

[B52] Zhou H. Lin-Wang K. Wang H. Gu C. Dare A. P. Espley R. V. (2015). Molecular genetics of blood-fleshed peach reveals activation of anthocyanin biosynthesis by NAC transcription factors. *Plant J.* 82 105–121. 10.1111/tpj.12792 25688923

