# Supplementary figures and images for: The SPB-Box Transcription Factor AaSPL2 Positively Regulates Artemisinin Biosynthesis in Artemisia annua L
Source: Front Plant Sci. 2019 Apr 9;10:409. doi: 10.3389/fpls.2019.00409 (PMC6465324; doi:10.3389/fpls.2019.00409)

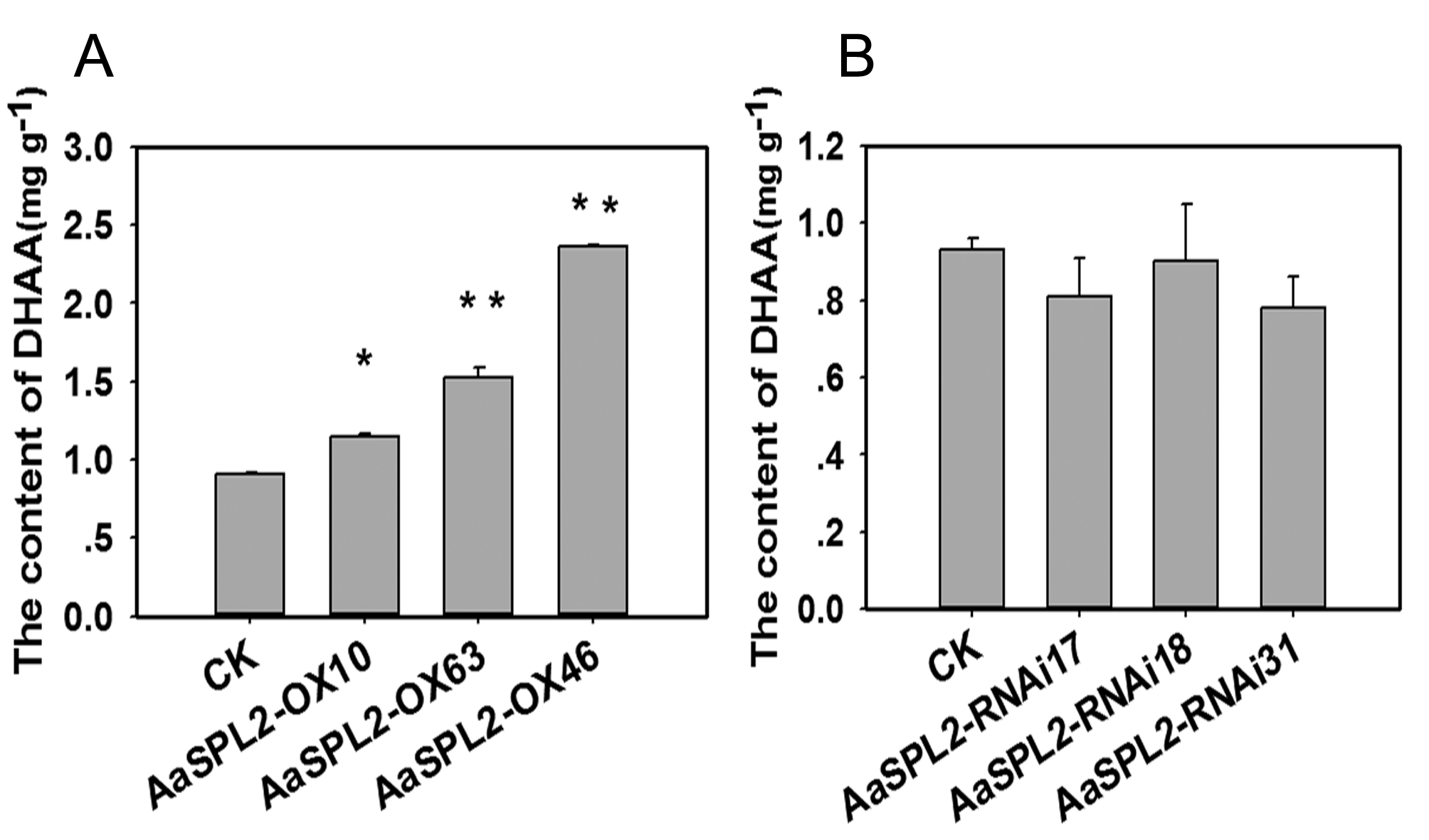

Supplement: FIGURE S1 — DHAA content in the transgenic plants was determined by HPLC. (A) DHAA content was detected in the AaSPL2 overexpression plants. (B) DHAA content was detected in the AaSPL2 RNAi plants. Error bars indicate SD (n = 3). ∗∗p < 0.01. [file Image_1.TIF]

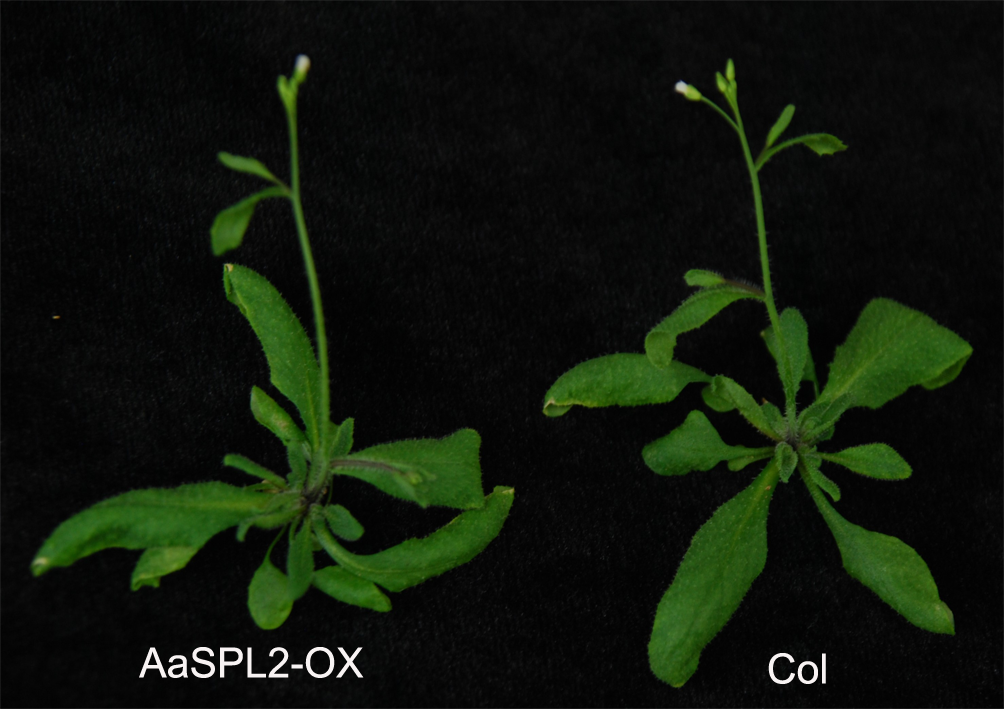

Supplement: FIGURE S2 — The phenotype of AaSPL2 overexpressed in Arabidopsis. [file Image_2.TIF]

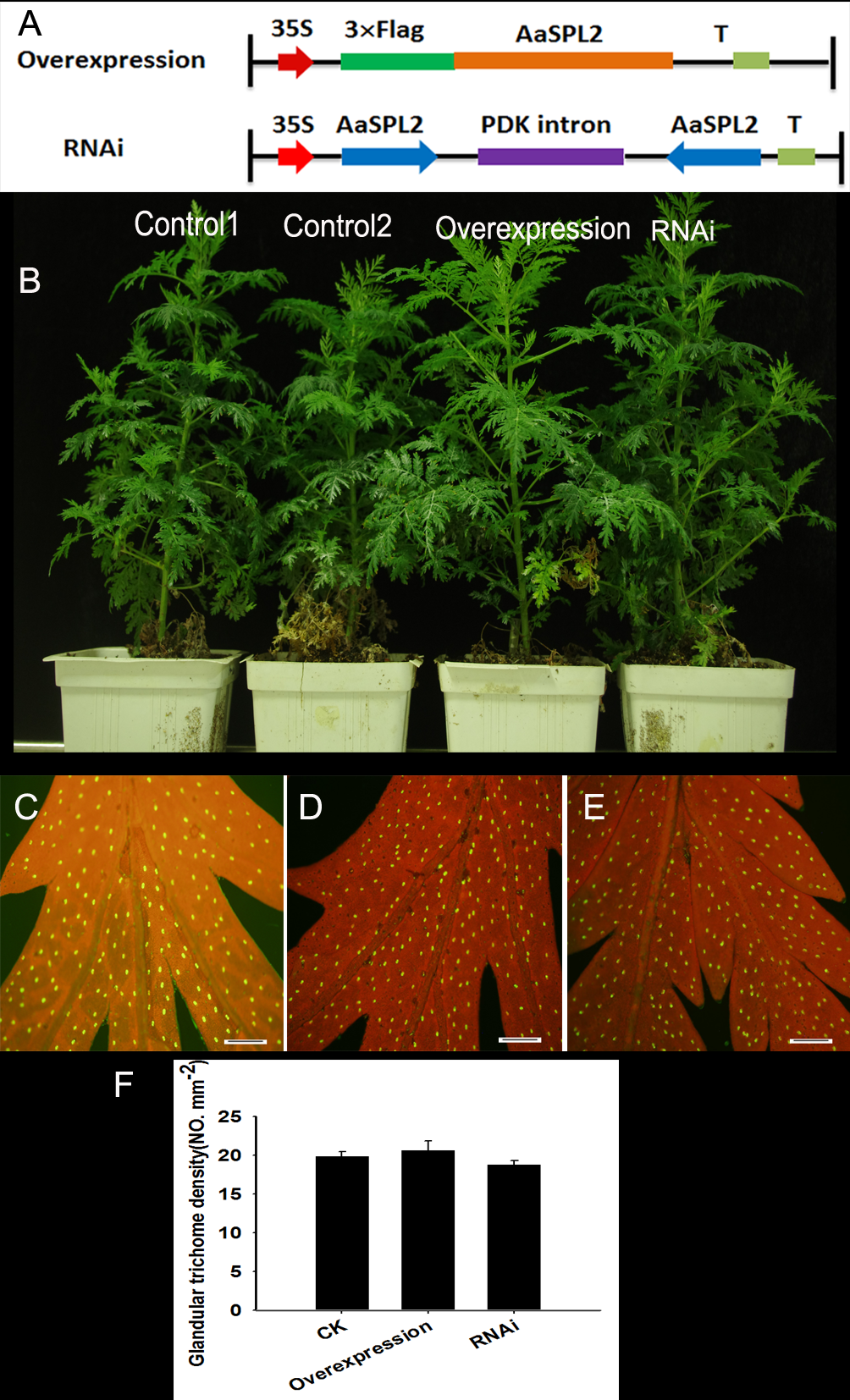

Supplement: FIGURE S3 — The phenotype of AaSPL2 overexpressed in A. annua. (A) A schematic representation of overexpression vector and RNAi vector used in the A. annua transformation is shown. (B) the phenotype of overexpression and RNAi transgenic plants. (C) Glandular trichome of control. (D) Glandular trichome of overexpression plants. (E) Glandular trichome of RNAi plants. (F) Trichome number of A. annua. Scale bar represents 0.5 mm. [file Image_3.TIF]
