# Supplementary material for: The SPB-Box Transcription Factor AaSPL2 Positively Regulates Artemisinin Biosynthesis in Artemisia annua L
Source: Front Plant Sci. 2019 Apr 9;10:409. doi: 10.3389/fpls.2019.00409 (PMC6465324; doi:10.3389/fpls.2019.00409)
Supplement: TABLE S1 — Primers used in this study. [file Table_1.docx]

Table S1 Primers used in this study

| Primers name | Primers sequence |
| --- | --- |
| Q-ALDH1-PF | GGACTTGCCTCAGGTGTAT |
| Q-ALDH1-PR | GTGCCTCTAATCCTTGTTC |
| Q-ADS-PF | GGACTAGGTTCAGGCTATG |
| Q-ADS-PR | AAGCATGTAATTGACCACC |
| Q-CYP-PF | TCATTTCAGTCGCTT |
| Q-CYP-PR | CCAGTTTGCCTCAGTA |
| Q-DBR2-PF | ACTGCTGGTGGCTTTCTTA |
| Q- DBR2-PR | ACCCTCGACTTGTTCCTTA |
| Q-Actin-PF | CCAGGCTGTTCAGTCTCTGTAT |
| Q-Actin-PR | CGCTCGGTAAGGATCTTCATCA |
| 3×pDBR -F | TCGAG TGTAGTACTTTTTGTAGTACTTTTTGTAGTACTTTTC |
| 3×pDBR -R | TCGAGAAAGTACTACAAAAAGTACTACAAAAAGTACTACA C |
| AaSPL2-F  AaSPL2-R  AaSPL2-F-RNAi  AaSPL2-R- RNAi | ATGGAGTGGAATTGGGACAA  TTAATTTGAACAAAAGTAGT  caccATGGAGTGGAATTGGGACAA  CTCAACTTGACAACGTGGCG |
